# Analysis of chemoresistance characteristics and prognostic relevance of postoperative gemcitabine adjuvant chemotherapy in pancreatic cancer

**DOI:** 10.1002/cam4.7229

**Published:** 2024-05-02

**Authors:** Jiaqiang Ren, Shuai Wu, Tong Su, Jiachun Ding, Fan Chen, Jie Li, Zheng Wang, Liang Han, Zheng Wu

**Affiliations:** ^1^ Department of Hepatobiliary Surgery The First Affiliated Hospital of Xi'an Jiaotong University Xi'an Shaanxi China; ^2^ School of Public Health, Xi'an Jiaotong University Health Science Center Xi'an Shaanxi China

**Keywords:** chemoresistance, gemcitabine, pancreatic cancer, prognosis, tumor size

## Abstract

**Aim:**

To investigate the relationship between chemoresistance in pancreatic cancer patients receiving postoperative gemcitabine adjuvant therapy and specific clinical/pathological characteristics, as well as its impact on patient prognosis.

**Methods:**

From June 2018 to June 2021, clinical and pathological data of 148 pancreatic cancer patients were collected, and 101 patients were followed up for tumor recurrence/metastasis and survival status. The correlation between chemoresistance and specific clinical/pathological characteristics or patient prognosis was retrospectively analyzed.

**Results:**

Of the 148 patients, 78 were in the chemoresistance group and 70 in the non‐chemoresistance group. Univariate analysis showed that the development of chemoresistance may be related to patient age, combined diabetes, preoperative CA19‐9 level, tumor size, AJCC stage, vascular invasion, and positive lymph node ratio. Furthermore, subsequent multivariate analysis incorporating these variables indicated that tumor size may be a key factor influencing chemoresistance (*p* < 0.001, OR = 1.584). Log‐rank test showed patients in the chemoresistance group had worse overall survival (OS) (HR = 2.102, *p* = 0.018) and progression free survival (PFS) (HR = 3.208, *p* = 0.002) than patients in the non‐chemoresistance group; and patients with smaller size tumors (diameter ≤3 cm) had significantly better OS (HR = 2.923, *p* < 0.001) and PFS (HR = 2.930, *p* = 0.003) than those with larger size tumors (diameter >3 cm).

**Conclusions:**

Patients with pancreatic cancer receiving postoperative gemcitabine adjuvant therapy are more likely to develop chemoresistance when their tumor sizes are larger (diameter >3 cm). Development of chemoresistance exacerbates the prognosis of patients with pancreatic cancer, and larger tumor size is also a risk factor for poor prognosis in these patients.

## 1 INTRODUCTION

Pancreatic cancer is a highly aggressive solid tumor that accounts for approximately 90% of all pancreatic malignancies.^1^ According to the most recent statistics,^2^ there were 62,210 new cases of pancreatic cancer in the United States in recent years, with approximately 49,830 deaths, making it the third leading cause of cancer death in the United States by 2022. Because the disease often lacks characteristic early manifestations, approximately 80% of patients with pancreatic cancer are often diagnosed at a late stage and are unable to undergo radical resection.

Chemotherapy has become a critical component of the current treatment of pancreatic cancer at all stages due to the high likelihood of local recurrence and metastasis in the majority of cases. Adjuvant chemotherapy after radical resection can significantly improve patients' disease‐free survival (DFS) and overall survival (OS). According to recent studies,^3^, ^4^ patients with limited pancreatic cancer who underwent radical pancreatic tumor resection had a median OS of 10–21 months, which was comparable to patients who did not receive the procedure; however, postoperative systemic chemotherapy significantly increased OS by up to 45 months.^4^

Gemcitabine was approved by the United States Food and Drug Administration (FDA) in 1996 and quickly became the standard of care for patients with all stages of pancreatic cancer,^5^ leading to significant improvements in treatment outcomes. Combination regimens of gemcitabine‐based chemotherapy, such as albumin‐bound paclitaxel plus gemcitabine,^6^ have been shown to improve overall survival in patients with metastatic pancreatic cancer to a limited extent. However, the chemoresistance during gemcitabine treatment severely limits its efficacy, and clinical control rates do not translate into improved overall prognosis. In addition, pancreatic cancer tends to be more resistant to gemcitabine than to other chemotherapeutic agents.

In patients with pancreatic cancer treated with gemcitabine as postoperative adjuvant therapy, many are forced to discontinue or change to other chemotherapy regimens due to chemoresistance, while some patients have good a response and can achieve long‐term control without progression. It remains unknown whether the development of chemoresistance is related to specific clinical/pathological characteristics of pancreatic cancer patients, or to the chemotherapy itself.

Since there were no previous studies on this topic, we designed this retrospective study to investigate the current status of chemoresistance in pancreatic cancer patients treated with gemcitabine as postoperative adjuvant therapy, its association with specific clinical/pathological characteristics, and its impact on patient prognosis. By identifying the possible characteristics of pancreatic cancer patients who are likely to develop chemoresistance, we can early detect the subgroup of patients who respond well to gemcitabine therapy. This will enable the provision of alternative chemotherapy regimens at an earlier stage, reducing delays in treatment and optimizing patient outcomes.

## 2 PATIENTS AND METHODS

A single‐center retrospective clinical study was designed to determine whether postoperative pancreatic cancer patients who are resistant to gemcitabine adjuvant chemotherapy share the same clinical/pathological characteristics. The study was reviewed and approved by the Ethics Review Committee of the First Affiliated Hospital of Xi'an Jiaotong University.

### 2.1 Patients

Clinical, imaging and pathological data were collected from patients diagnosed with pancreatic cancer at the First Affiliated Hospital of Xi'an Jiaotong University between June 1st, 2018 and June 1st, 2021. All clinical data were obtained from the hospital's current operating medical record system, imaging data were obtained from the hospital's picture archiving and communication system (PACS), and pathological data were obtained from pathology reports issued by pathologists. To ensure data reliability, all patient data were collected independently by two investigators and randomly cross‐validated, and then collated simultaneously by other two investigators at the data collation stage and rechecked for outliers and anomalous data.

Clinical and pathological data included: (1) Baseline information such as patient ID, name, gender, age at diagnosis, body mass index (BMI), whether hypertension and diabetes were combined (“combined hypertension” indicates the diagnosis of pancreatic cancer patients with concomitant hypertension; “combined diabetes” indicates the diagnosis of pancreatic cancer patients with concomitant diabetes mellitus), and preoperative and postoperative carbohydrate antigen 19‐9 (CA19‐9) levels (U/mL); (2) Surgical and pathological information such as surgical procedure, tumor diameter, American Joint Committee on Cancer (AJCC) staging (8th edition), whether extrapancreatic invasion, lymph node metastasis, perineural invasion, and vascular invasion were combined, the total number of lymph nodes detected, number of positive lymph nodes, and positive lymph node ratio; (3) Chemotherapy information such as chemotherapy modality, chemotherapy regimen, and chemotherapy cycle; (4) Follow‐up information such as overall survival (OS) and progression free survival (PFS). After the initial inclusion of all patients with pancreatic cancer, the exclusion criteria were as follows: (1) clinically confirmed diagnosis without pathological evidence, (2) other pathological types of non‐pancreatic ductal adenocarcinoma, (3) not receive chemotherapy after diagnosis or chemotherapy regimen that did not include gemcitabine, (4) patients receiving preoperative adjuvant therapy, and (5) chemotherapy failure due to physical intolerance.

### 2.2 Definition and grouping

The endpoint of the study was the development of gemcitabine chemoresistance during treatment. According to Response Evaluation Criteria in Solid Tumors (RECIST) version 1.17, systematic assessment focusing on the target lesion is recommended at the end of every two treatment cycles after cancer patients starting treatment.^7^ A 1‐month gemcitabine treatment cycle consists of 3 weeks of weekly treatment followed by 1 week off. Since the mean OS for patients with all stages of pancreatic cancer (including pancreatic resection patients) is approximately 12.6 months,^8^ the median value of mean OS of 6 months (6 cycles) is used to determine whether the treatment is effective.

**FIGURE 1:**
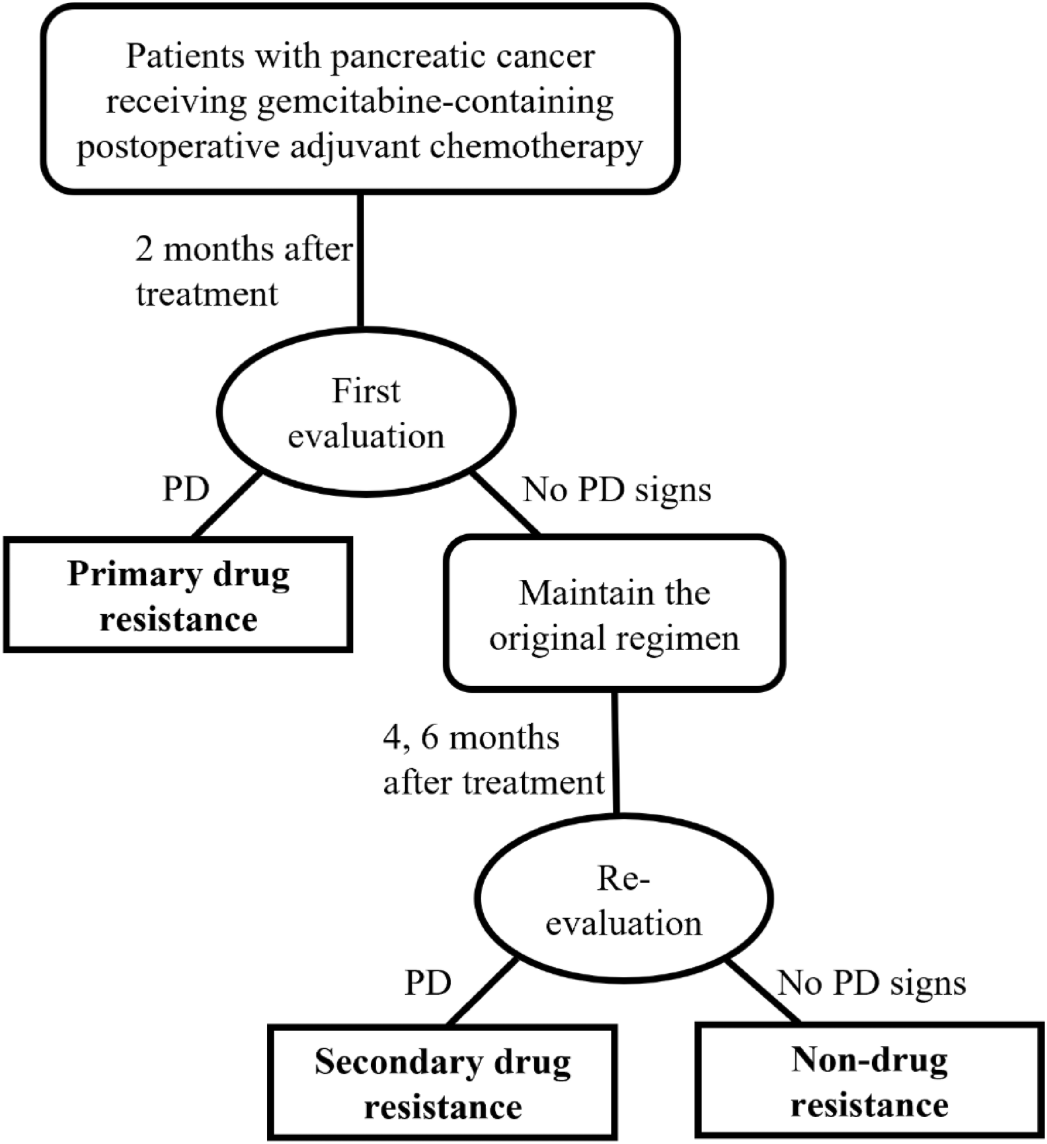
Screening procedure for gemcitabine‐resistant pancreatic cancer patients. PD, progressive disease.

According to RECIST, the criteria of progressive disease (PD) in patients who receive postoperative adjuvant therapy after radical surgery were: significant lesions reappear after completely regress, this holds true for all types of metastases. If the treatment was previously considered to be completely effective, then the reappearance of any lesion, or the development of a new lesion (including a new lymph node that meets the size criteria for a pathologic lymph node) should be considered PD. And chemoresistance to gemcitabine was defined as PD assessed within 6 months of starting chemotherapy with the gemcitabine regimen, while assessment of no PD signs was considered non‐chemoresistance, that is, treatment effective.^9^ Patients in the drug resistance group were further classified as having primary or secondary resistance (Figure 1).

**FIGURE 2:**
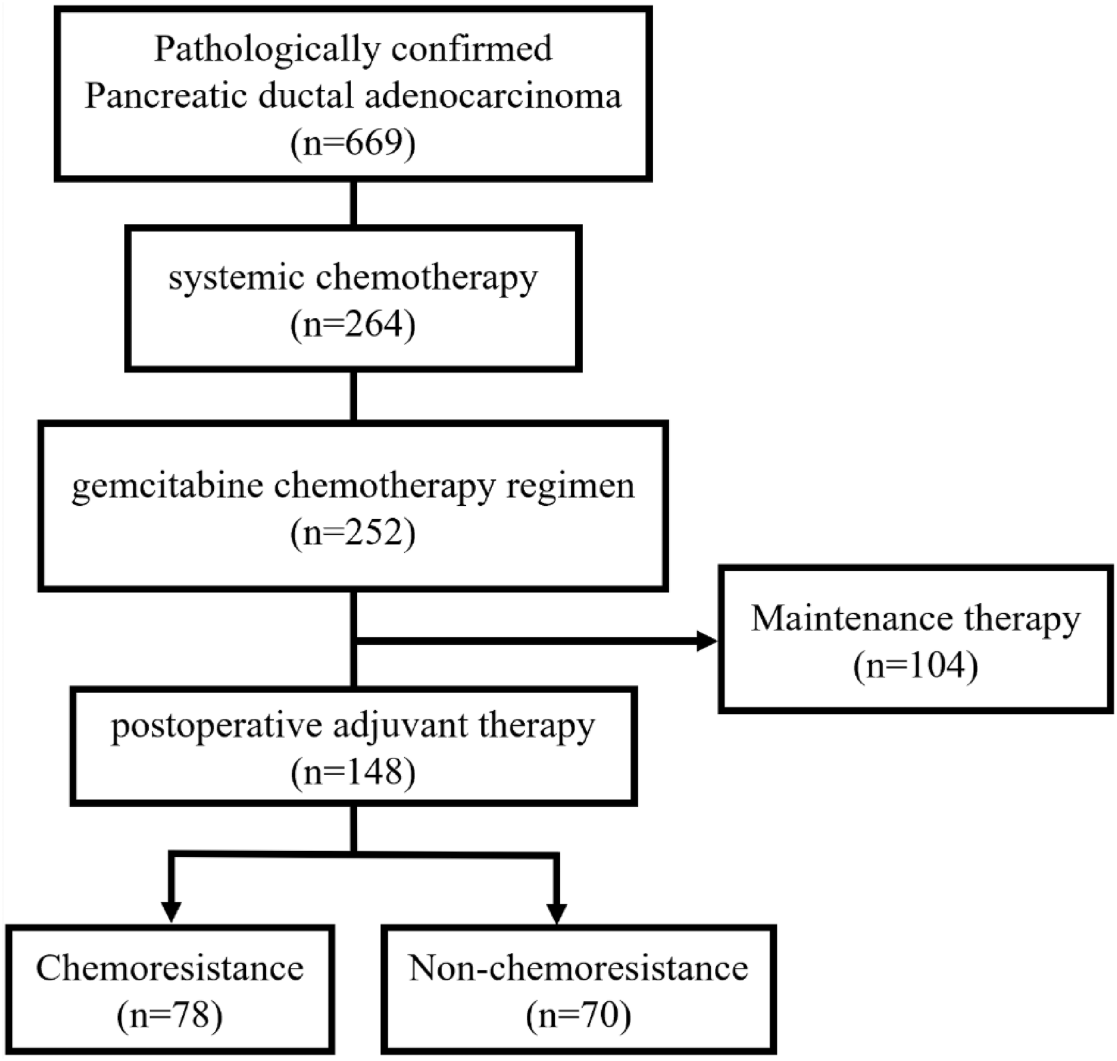
Flow diagram of inclusion in the study.

After screening, a total of 148 pancreatic cancer patients were enrolled in the final study and divided into two groups, 78 in the chemoresistance group and 70 in the non‐chemoresistance group (Figure 2).

### 2.3 Statistical analysis

Descriptive statistics were used to describe the distribution of clinical/pathological characteristics among all patients, with measures described as mean standard deviation if normally distributed and median (range) if skewed. Count data were described as a ratio of the proportion of individuals in the group. The Pearson chi‐squared test or Fisher's exact test was used to compare categorical variables between groups; the Student's *t*‐test was used to compare continuous variables that followed a normal distribution, and the nonparametric Mann–Whitney *U* test was used when they did not follow to a normal distribution.

Multivariate logistic regression models were used to test predictors associated with outcome variables. The odds ratio (OR) and 95% confidence interval (CI) were calculated and presented in forest plots. OS and PFS were assessed using Kaplan–Meier curve analysis and the Log‐rank (Mantel–Cox) test. For the two‐tailed tests, a *p* value<0.05 was considered as statistically significant. All statistical analyses were performed with SPSS version 21.0 (Inc., Chicago, IL, USA), and all graphs were generated with GraphPad Prism 9.4 (Inc., CA).

## 3 RESULTS

### 3.1 Statistics of chemoresistance‐related events

The occurrence of chemoresistance‐related events indicates disease progression during treatment. We counted the chemoresistance events for each patient using RECIST 1.1 criteria and found that: cancer metastasis was the most common event of disease progression (89.7%). And consistent with previous studies, the liver (38.5%) was the most common site of metastasis in pancreatic cancer, followed by abdominal lymph node metastasis (23.1%) and lung metastasis (11.5%), among others. (Table 1).

**TABLE 1:** Statistics of progressive events in the chemoresistance group patients receiving postoperative adjuvant chemotherapy.

Progression events	Number	Percentage
Tumor recurrence	8	10.3%
Tumor metastasis		
Liver	30	38.5%
Lung	9	11.5%
Bone	7	9.0%
Abdominal lymph nodes	18	23.1%
Other sites	6	7.7%

### 3.2 Baseline characteristics

In all patients, the male to female ratio was approximately 1.3:1. We found that the median age of patients in the chemoresistance group was higher than that of patients in the non‐chemoresistance group (61 vs. 57 years, *p* = 0.008) with a statistically significant difference (Table 2). There were significantly more patients in the chemoresistance group with combined diabetes (32.1% vs. 17.1%, *p* = 0.037) than in the non‐chemoresistance group, but no difference with combined hypertension. Meanwhile, our results showed significantly higher levels of the preoperative tumor marker CA19‐9 (163.15 vs. 66.53 U/mL, *p* = 0.027) in the chemoresistance group compared with the non‐chemoresistance group, but no significant difference with postoperative levels.

**TABLE 2:** Baseline characteristics of patients between the chemoresistance and non‐chemoresistance group.

Characteristics	Chemoresistance, No. (column%/rows%)		Statistic (χ^2^/*t*/*Z*)	*p*‐value
	Yes (*n* = 78)	No (*n* = 70)		
Sex			0.334	0.563
Male	42 (53.8/50.6)	41 (58.6/49.4)		
Female	36 (46.2/55.4)	29 (41.4/44.6)		
Age at diagnosis, mean ± SD	60.96 ± 8.76	56.89 ± 9.61	2.699	0.008
BMI, mean ± SD	22.32 ± 2.91	22.30 ± 3.10	0.047	0.963
Hypertension	16 (20.5/53.3)	14 (20.0/46.7)	0.006	0.938
Diabetes	25 (32.1/67.6)	12 (17.1/32.4)	4.373	0.037
CA 19‐9, U/mL, median (range)				
Before the surgery	163.15 (0.89–10000.00)	66.53 (0.60–1454.62)	−2.216	0.027
After the surgery	31.60 (0.60–10000.00)	22.30 (0.60–1178.00)	−0.275	0.784

Abbreviations: BMI, body mass index, kg/m^2^; CA 19‐9, carbohydrate antigen 19‐9.

### 3.3 Surgical and pathological characteristics

In this study, patients who underwent pancreaticoduodenectomy (PDT, 60.8%) became the most common radical pancreatectomy (Table 3). More importantly, we found that the median tumor size of patients in the chemoresistance group was significantly larger (4.00 vs. 2.50 cm, *p* < 0.001) than that in the non‐chemoresistance group; after further differentiating all patients using 3 cm (the overall median tumor diameter) as the tumor cut‐off value, we concluded that patients in the chemoresistance group were prone (65.4%) to have tumors >3 cm in diameter, whereas tumors in the non‐chemoresistance group were more likely (64.9%) to be ≤3 cm in diameter, with statistically significant differences (*p* < 0.001).

**TABLE 3:** Surgical and pathological characteristics of patients between the chemoresistance and non‐chemoresistance group.

Characteristics	Chemoresistance, No. (column%/rows%)		Statistic (*χ* ^2^/*Z*)	*p*‐value
	Yes (*n* = 78)	No (*n* = 70)		
Technique^a^			5.049	0.070
PDT^a^	50 (64.1/33.8)	40 (57.1/44.4)		
DP^a^	22 (28.2/43.1)	29 (41.4/56.9)		
TP^a^	6 (7.7/85.7)	1 (1.4/14.3)		
Tumor size^a^, cm, median (range)	4.00 (1.30–8.50)	2.50 (0.50–8.00)	−5.408	<0.001
≤3 cm	27 (34.6/35.1)	50 (71.4/64.9)	20.031	<0.001
>3 cm	51 (65.4/71.8)	20 (28.6/28.2)		
AJCC stage			4.057	0.044
≤IIA^a^	35 (44.9/44.9)	43 (61.4/55.1)		
≥IIB^a^	43 (55.1/61.4)	27 (38.6/38.6)		
Extrapancreatic invasion			0.053	0.819
Positive	70 (89.7/53.0)	62 (88.6/47.0)		
Negative	8 (10.3/50.0)	8 (11.4/50.0)		
Lymph node metastasis			2.633	0.105
Positive	37 (47.4/60.7)	24 (34.3/39.3)		
Negative	41 (52.6/47.1)	46 (65.7/52.9)		
Vascular invasion			4.065	0.044
Positive	36 (46.2/63.2)	21 (30.0/36.8)		
Negative	42 (53.8/46.2)	49 (70.0/53.8)		
Perineural invasion			0.035	0.851
Positive	66 (84.6/52.4)	60 (85.7/47.6)		
Negative	12 (15.4/54.5)	10 (14.3/45.5)		
Total number of lymph nodes, mean ± SD	13.00 ± 9.66	12.46 ± 9.00	−0.506	0.613
Positive lymph nodes, mean ± SD	1.27 ± 2.50	0.69 ± 1.11	−1.595	0.111
Positive ratio, mean ± SD	0.09 ± 0.14	0.06 ± 0.12	−2.042	0.041

^a^ For patients who underwent multiple radical surgeries, only the first surgery was counted in the statistics; The tumor size was included in the statistics with the longest diameter measured by the tumor; PDT, pancreaticoduodenectomy; DP, distal pancreatectomy; TP, total pancreatectomy; ≤2A group, includes patients with AJCC stages IA, IB, and IIA; ≥2B group, includes patients with AJCC stages IIB, 3, and 4.

After evaluating each patient strictly according to the latest AJCC staging guidelines, we divided all patients into two groups (≤2A, including stages IA, IB, IIA; ≥2B group, including stages IIB, 3, 4) for analysis. we found that patients in the chemoresistance group tended to have a combination of a later stage. In addition, statistical analysis of the tumor pathology showed that patients in the chemoresistance group were more likely to have vascular invasion compared to the non‐chemoresistance group, and the difference was statistically significant (63.2% vs. 36.8%, *p* = 0.044).

### 3.4 Chemotherapy‐related characteristics

Chemotherapy alone was the most common treatment modality (80.8%, 85.7%) for patients in both groups (Table 4), followed by chemotherapy plus targeted therapy. Regarding the chemotherapy regimen, the regimen of gemcitabine plus albumin‐combined paclitaxel (AG) was the most commonly used (54.7%), followed by the regimen of gemcitabine plus S‐1 (GS) (37.8%). Statistical analysis showed no statistically significant difference in the distribution of treatment modality and chemotherapy regimen between the two groups. The mean chemotherapy cycles were significantly much longer in the non‐chemoresistance group (7.13 vs. 2.90 months, *p* < 0.001) than in the chemoresistance group due to the study design.

**TABLE 4:** Chemotherapy‐related characteristics of patients between the chemoresistance and non‐chemoresistance group.

Characteristics	Chemoresistance, No. (column%) (rows%)		Statistic (*χ* ^2^/*Z*)	*p*‐value
	Yes (*n* = 78)	No (*n* = 70)		
Treatment modality			2.553	0.713
Chemotherapy	63 (80.8/51.2)	60 (85.7/48.8)		
Chemotherapy plus radiotherapy	6 (7.7/75.0)	2 (2.9/25.0)		
Chemotherapy plus targeted therapy	7 (9.0/58.3)	5 (7.1/41.7)		
Chemotherapy plus radiotherapy plus targeted therapy	1 (1.3/50.0)	1 (1.4/50.0)		
Chemotherapy plus other therapy^a^	1 (1.3/33.3)	2 (2.9/66.7)		
Chemotherapy regimen			4.897	0.413
AG^a^	40 (51.3/49.4)	41 (58.6/50.6)		
GS^a^	30 (38.5/53.6)	26 (37.1/46.4)		
GX^a^	2 (2.6/50.0)	2 (2.9/50.0)		
GEMOX^a^	1 (1.3/50.0)	1 (1.4/50.0)		
GP^a^	1 (1.3/100.0)	0		
G^a^	4 (5.1/100.0)	0		
Chemotherapy cycle, mean ± SD	2.90 ± 1.30	7.13 ± 2.01	−10.571	<0.001

^a^ Other therapies include perfusion therapy, immunotherapy, interventional therapy, etc. AG, gemcitabine plus albumin‐bound paclitaxel; G, gemcitabine monotherapy; GEMOX, gemcitabine plus oxaliplatin; GP, gemcitabine plus cisplatin; GS, gemcitabine plus S‐1; GX, gemcitabine plus capecitabine.

### 3.5 Multivariate regression analysis

**FIGURE 3:**
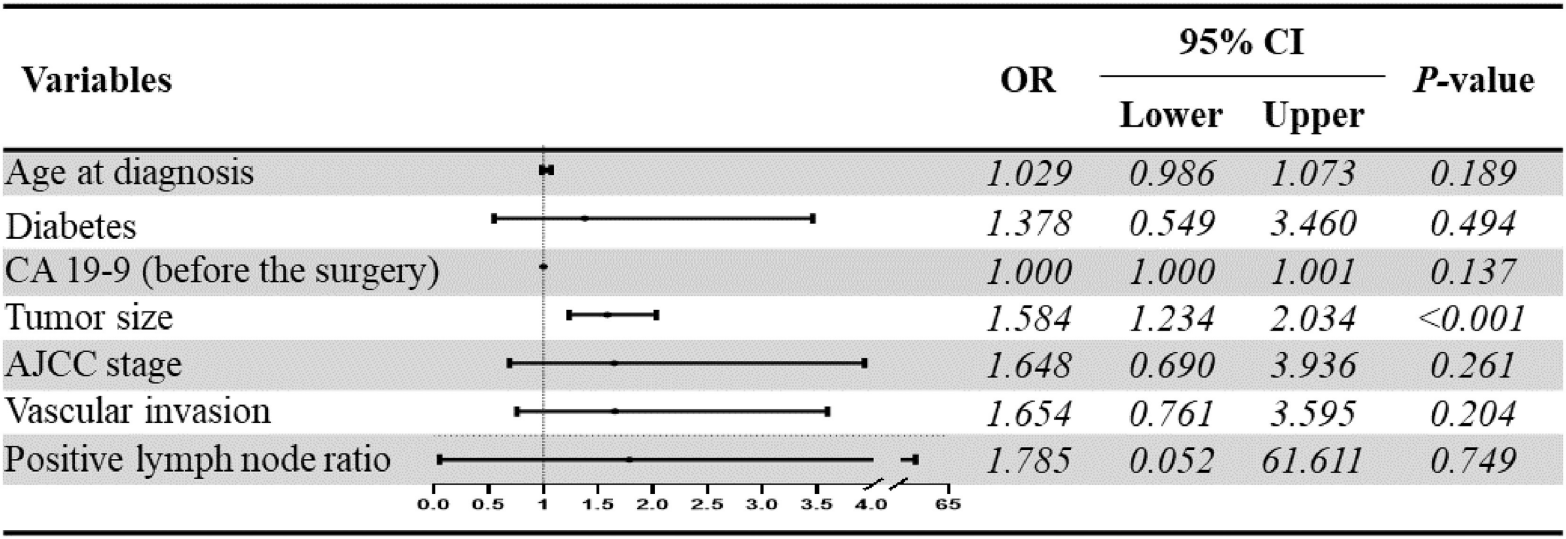
Multivariate regression analysis of chemoresistance‐related variables.

In the univariate analysis, we found that the distribution of characteristics such as age at diagnosis, diabetes, CA 19‐9 (before the surgery), tumor size, AJCC stage, vascular invasion, and positive lymph node ratio was statistically different between groups (*p* < 0.05), and to exclude mutual interference between these variables, we constructed a multivariate analysis model using binary logistic regression (Figure 3). The model was validated for better fit (*R* ^2^ = 0.032), multicollinearity between variables was excluded (VIF <10) and predictive accuracy was good (70.3%).

The results of our analysis suggest that tumor size may be a key factor influencing chemoresistance, with the odds of chemoresistance increasing with increasing tumor size (*p* < 0.001, OR = 1.584). The other variables in the logistic regression analysis did not show significant values (*p* > 0.05).

### 3.6 Prognosis and its relationship with chemoresistance

**FIGURE 4:**
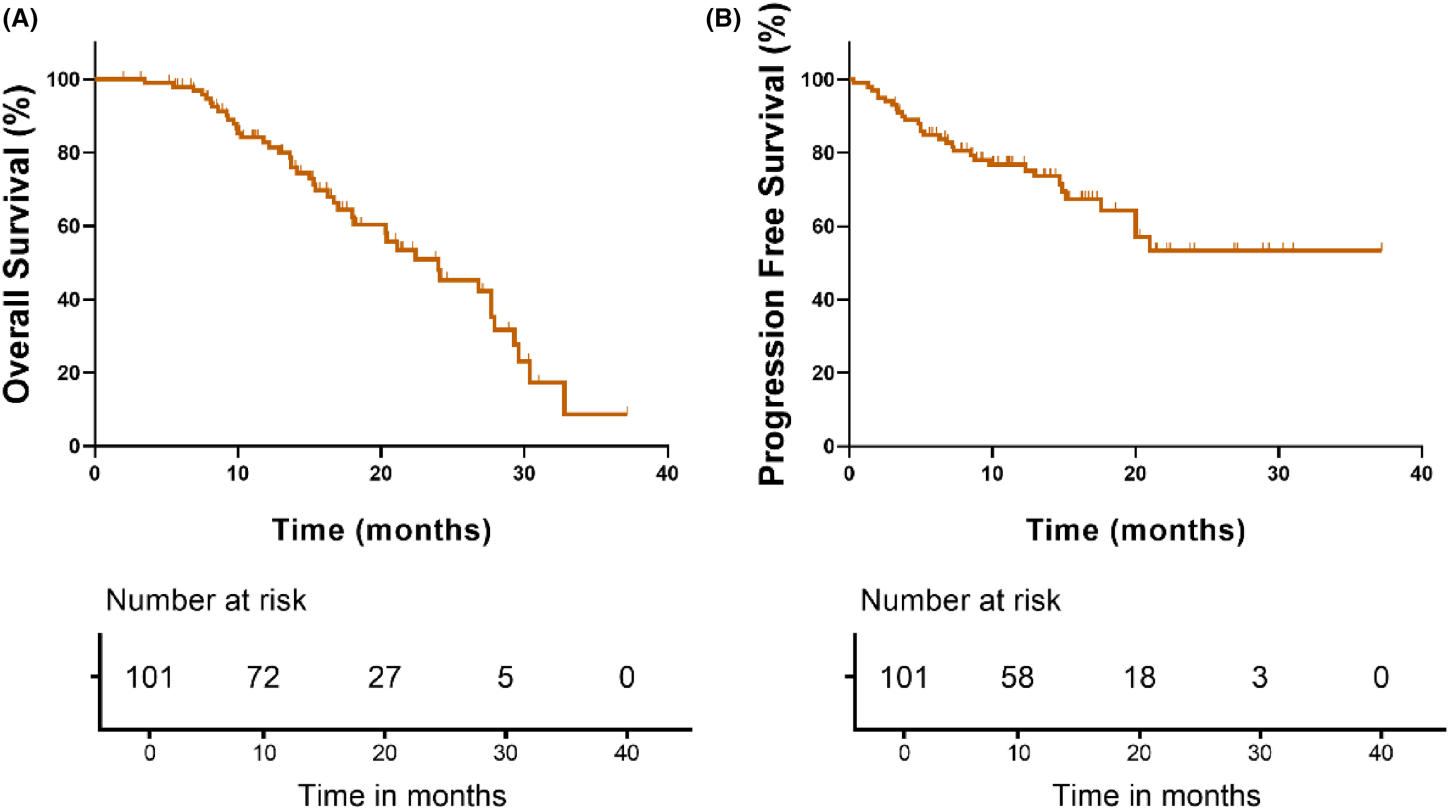
Kaplan–Meier survival curve by OS (A) and PFS (B) of all patients in the adjuvant therapy group. OS, overall survival, means the time from the start of diagnosis to death from any cause; PFS, progression free survival, means the time from the start of diagnosis to the onset of any aspect of tumor progression.

In our study, a total of 101 (68.2%) patients were completed for follow‐up on tumor recurrence/metastasis and survival status. The follow‐up period ended on December 1st, 2021, and the median follow‐up time was 13.4 months (0.3–37.2). In all 101 patients, the range of OS was 2.0–37.2 months, while PFS was 0.3–37.2 months (Figure 4).

**FIGURE 5:**
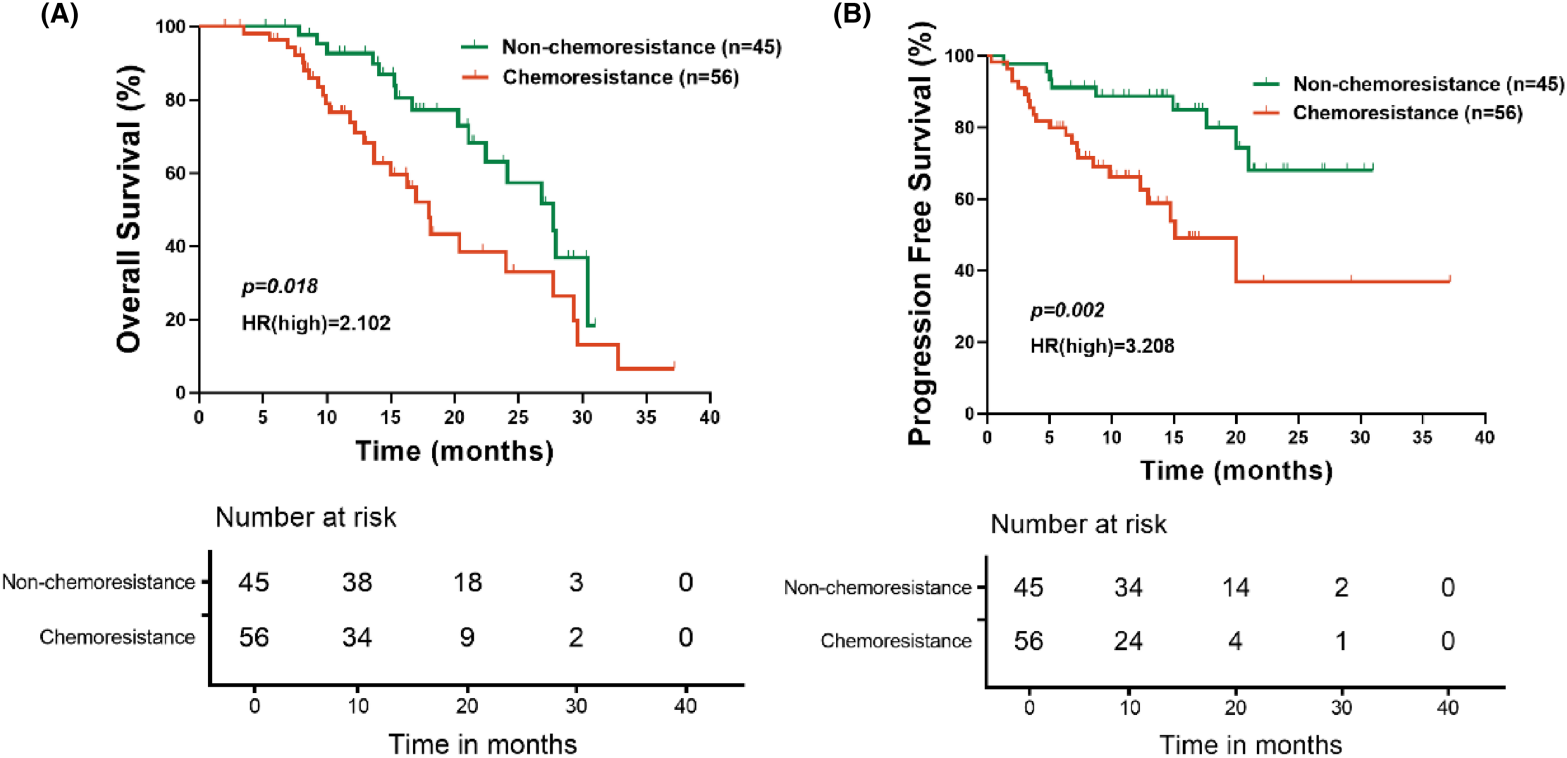
Log‐rank test by OS (A) and PFS (B) of patients between the chemoresistance and non‐chemoresistance groups.

We then analyzed the prognosis of patients in the chemoresistance and non‐chemoresistance groups separately and found a clear difference between the two groups. The OS (HR = 2.102, *p* = 0.018) and PFS (HR = 3.208, *p* = 0.002) of patients in the non‐chemoresistance group were significantly better than those in the chemoresistance group, with statistically significant differences (Figure 5).

### 3.7 The relationship between tumor size and prognosis

Based on the results of univariate and multivariate analysis, we found that tumor size may be a key factor influencing the phenomenon of chemoresistance in postoperative gemcitabine adjuvant therapy of pancreatic cancer patients, so further analysis was designed to find out its relationship with the prognosis of these patients.

**FIGURE 6:**
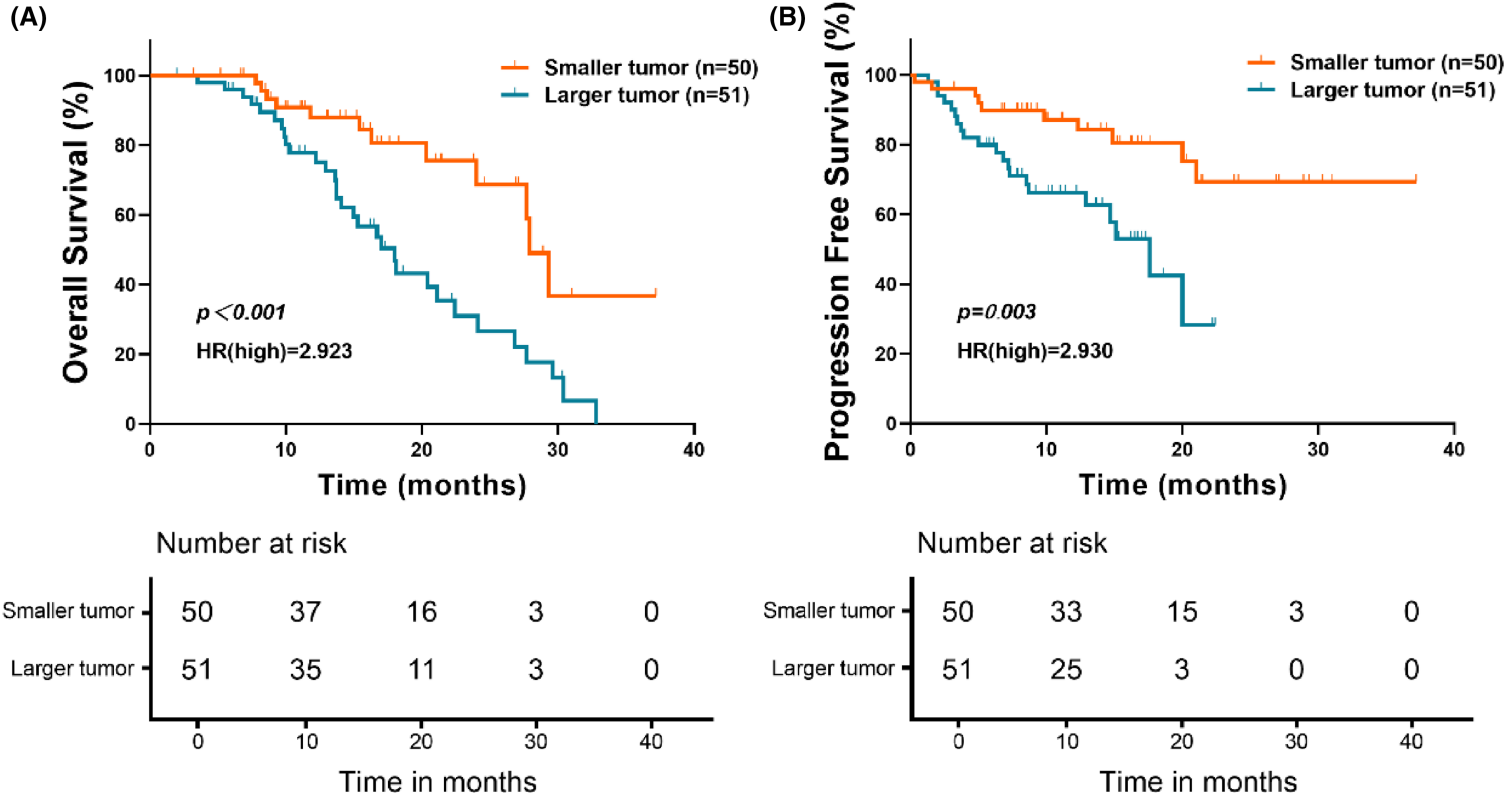
Log‐rank test by OS (A) and PFS (B) of the patients with different tumor sizes.

Log‐rank test results showed that patients with smaller tumors (diameter ≤3 cm) had significantly longer OS (HR = 2.923, *p* < 0.001) and PFS (HR = 2.930, *p* = 0.003) compared with those with larger tumors (diameter >3 cm) (Figure 6).

## 4 DISCUSSION

Pancreatic cancer is a highly malignant disease that is a leading cause of cancer death globally with a very poor prognosis. Although early surgical resection is currently the only potentially curative option for this solid tumor, it is only available to less than 20% of patients at the time of diagnosis. Even after surgery, the majority of patients experience recurrence and metastasis.^10^ As a result, chemotherapy is by far the most effective treatment for pancreatic cancer. Gemcitabine is used as the standard of care in pancreatic cancer chemotherapy regimens, but it is often ineffective due to the development of chemoresistance during treatment.

The purpose of this retrospective study was to determine whether the phenomenon of chemoresistance that develops during the treatment of pancreatic cancer patients with postoperative gemcitabine adjuvant therapy is associated with specific clinical characteristics. Patients were divided into chemoresistance and non‐chemoresistance groups based on their response to chemotherapy, and the results were obtained by statistical analysis. Of the 148 pancreatic cancer patients finally included in the study, 78 patients (52.7%) developed chemoresistance, indicating that only a small proportion of patients were effectively treated with the gemcitabine regimen. Our results are consistent with previous review articles,^11^, ^12^ showing that the development of gemcitabine resistance severely limits the efficacy of this chemotherapy.

According to previous studies, distant metastasis of cancer cells is the most common manifestation of chemotherapy resistance in pancreatic cancer patients. The assessment of disease as PD during treatment is a confirmation of gemcitabine chemoresistance, which may manifest as tumor recurrence after radical resection or distant metastasis of cancer cells. According to previous studies,^13^ the most common metastatic sites of advanced pancreatic cancer are the liver (90%), lymph nodes (25%), lung (25%), peritoneum (20%), and bone (10%–15%). Our findings followed a similar pattern, with liver metastasis (32.89%), abdominal lymph node metastasis (19.74%), and lung metastasis (10.53%) in descending order of prevalence. Therefore, it is evident that the liver serves as the principal site of metastasis in advanced pancreatic cancer.

Pancreatic cancer tends to present at a later median age. a study of 60,340 US patients with pancreatic cancer showed that the median age at diagnosis was 71 years.^14^ Our research showed that the mean age of patients with chemoresistance was higher than that of non‐chemoresistance patients, which was statistically significant. This suggests that older pancreatic cancer patients may face greater treatment difficulties due to chemoresistance, in addition to being less tolerant to chemotherapy due to deterioration in physical function. Previous research^15^ has linked an increase in BMI to the development of pancreatic cancer. And being overweight or obese in early adulthood is associated with a higher risk of pancreatic cancer and a younger age of onset, while obesity in old age is associated with poorer overall survival in pancreatic cancer patients. However, in our study, there was no significant difference in median BMI between chemoresistance and non‐chemoresistance patients.

Diabetes has been strongly associated with pancreatic cancer, and the results of a meta‐analysis of a total of 2408 pancreatic cancer patients by Liao et al^16^ showed a strong association (linear dose–response relationship) between fasting glucose concentration and pancreatic cancer incidence within the range of prodromal diabetes and diabetes, that is, for every 0.56 mmol/L increase in fasting glucose, the incidence of pancreatic cancer increased. As a result, we found that combined diabetes was more common in patients with chemoresistance than non‐chemoresistance patients, suggesting that glycemic control may play a more important role in pancreatic cancer.

CA19‐9 is a widely used biomarker for the diagnosis of pancreatic cancer. Pedro et al^17^ found that ROC curve analysis using baseline CA19‐9 levels with an area under the curve of 0.868 (95% CI, 0.782–0.954) and a cut‐off value of 2504 U/mL had a sensitivity of 100% and specificity of 82.8% for early death. In addition, CA19‐9 levels in advanced pancreatic adenocarcinoma are independently associated with poor prognosis and early death. We found that CA19‐9 levels before the surgery were significantly higher in the chemoresistance group than in the non‐chemoresistance group, and there was no significant difference in the distribution of postoperative CA19‐9 levels due to a significant decrease. It suggests that preoperative CA19‐9 levels may also be significant in indicating poor prognosis of pancreatic cancer patients.

In patients with pancreatic cancer, the size of the tumor and its relative position in the blood vessels usually determine whether the patient has the opportunity to undergo radical surgery. Interestingly, our study found that tumor size was not only significantly associated with chemoresistance (consistency of univariate and multivariate analysis), but may also affect the prognosis of patients with pancreatic cancer. This finding is consistent with that of Yeo et al. in their studies published in 1995^18^ and 1997,^19^ and more prospective studies are needed to confirm it. After dividing the patient AJCC staging by the median value, we found that pancreatic cancer patients with earlier AJCC staging seemed to be less prone to chemoresistance in treatment. Our study also revealed that patients with vascular invasion negative tumors tended to be non‐chemoresistance to gemcitabine, also the ratio of tumor lymph node metastasis was found to be significantly higher in the chemoresistance group than in the non‐chemoresistance group. These findings reinforce the importance of early diagnosis of pancreatic cancer patients and that early surgical resection with postoperative gemcitabine adjuvant chemotherapy provides a better prognosis for patients.

Several previous studies have explored combination regimens to enhance the efficacy of gemcitabine in treating pancreatic cancer. Stathis and Moore's phase III clinical trial study^20^ showed a significantly longer OS in the gemcitabine plus erlotinib (an EGFR inhibitor) treatment group compared to the gemcitabine alone group (6.2 months vs. 5.9 months, *p* = 0.038, HR = 0.82, 95% CI, 0.69–0.99) and a significantly longer DF in the combination group (23% vs. 17%; *p* = 0.023). The combination group also had a higher one‐year survival rate (23% vs 17%; *p* = 0.023) and significantly longer PFS (HR = 0.77, 95% CI, 0.64–0.92, *p* = 0.004). A study completed by Daniel et al^6^ on gemcitabine in combination with albumin‐bound showed that the albumin‐conjugated paclitaxel plus gemcitabine group had a higher median OS (8.5 months vs. 6.7 months, HR = 0.72, 95% CI, 0.62–0.83, *p* < 0.001), one‐year survival (35% vs. 22%), two‐year survival (9% vs. 4%), and median PFS (5.5 months vs. 3.7 months; HR = 0.69, 95% CI, 0.58–0.82, *p* < 0.001), and remission rate (23% vs 7%, *p* < 0.001). However, it is important to note that this combination led to elevated incidences of peripheral neuropathy and myelosuppression, contributing to the preference for incorporating gemcitabine into combination regimens in recent years.

Our results showed that chemotherapy regimens were dominated by combinations of two or more drugs, with only 3.4% of patients receiving gemcitabine monotherapy, supporting that chemotherapy combined with targeted therapy is gradually gaining popularity. We suspect that the widespread use of AG regimens is related to guideline updates, as the most recent National Comprehensive Cancer Network (NCCN) guidelines^21^ include AG regimens as a recommended treatment option for pancreatic cancer in a variety of settings, including locally advanced, borderline resectable, metastatic disease, and preoperative neoadjuvant. However, while the distribution of different types of treatment modalities and chemotherapy regimens differed between the two groups in our study, the differences were not statistically significant. One possible explanation is that sample size of the study was insufficient, and the resulting standard errors in the statistics provided insufficient power to detect statistical differences.

Of course, our study has limitations in the following areas: as a retrospective study, it was not possible to directly establish the causal relationship between the independent and dependent variables. For example, the factors identified in our study, such as larger tumor size and lymph node invasion, can directly affect the progression of pancreatic cancer and patient prognosis, while the development of chemoresistance also contributes to this outcome, so the exact causal relationship needs to be revealed by future prospective cohort studies with larger samples and multiple centers. Furthermore, there was also the unavoidable inclusion of some patients with incomplete data were included in the data collection (e.g., prognosis information was not available for some patients), which caused some difficulties in the subsequent statistical analysis.

In conclusion, our study focused for the first time on the clinical/pathological characteristics of pancreatic cancer patients who were resistant to postoperative gemcitabine adjuvant chemotherapy. We concluded that patients with pancreatic cancer are more likely to develop chemoresistance when their tumor sizes are larger (diameter >3 cm). Our analysis suggests that the development of chemoresistance exacerbates the prognosis of patients with pancreatic cancer, while larger tumor size is also a risk factor for the poor prognosis in these patients. In clinical practice, patients with pancreatic cancer should be diagnosed as early as possible, and patients with postoperative pancreatic cancer with larger tumor diameters may be considered for substitution with a more effective chemotherapy regimen other than gemcitabine.

## AUTHOR CONTRIBUTIONS

**Jiaqiang Ren:** Conceptualization (lead); data curation (lead); formal analysis (equal); investigation (lead); methodology (lead); resources (equal); writing – original draft (lead); writing – review and editing (equal). **Shuai Wu:** Conceptualization (equal); data curation (equal); investigation (equal); project administration (equal); supervision (equal); validation (equal); visualization (equal); writing – review and editing (supporting). **Tong Su:** Conceptualization (equal); data curation (equal); formal analysis (lead); investigation (equal); methodology (lead); software (lead); validation (equal); visualization (equal); writing – review and editing (supporting). **Jiachun Ding:** Data curation (equal); formal analysis (equal); methodology (equal); software (equal); validation (equal); visualization (equal). **Fan Chen:** Investigation (equal); methodology (equal); resources (equal); software (equal); visualization (equal). **Jie Li:** Conceptualization (equal); investigation (equal); supervision (equal); writing – review and editing (supporting). **Zheng Wang:** Funding acquisition (supporting); project administration (equal); supervision (equal); visualization (equal); writing – review and editing (equal). **Liang Han:** Conceptualization (equal); data curation (equal); formal analysis (equal); methodology (lead); project administration (equal); resources (equal); supervision (equal); writing – original draft (supporting); writing – review and editing (equal). **Zheng Wu:** Conceptualization (lead); funding acquisition (lead); project administration (lead); resources (lead); supervision (lead); writing – original draft (supporting); writing – review and editing (lead).

## FUNDING INFORMATION

This work was supported by grants from the National Natural Science Foundation of China (No. 82203756) and Department of Science and Technology of Shaanxi Province, China (No. 2022PT‐35) and the First Affiliated Hospital of Xi'an Jiaotong University (No. XJTU1AF‐CRF‐2022‐034).

## CONFLICT OF INTEREST STATEMENT

The authors declare no conflicts of interest.

## ETHICS STATEMENT

The Ethics Review Committee of the First Affiliated Hospital of Xi'an Jiaotong University approved this retrospective study. All methods in this study were conducted according to relevant guidelines and regulations, and informed consent was obtained from all patients included in the study.

## Data Availability

The data supporting the findings of this study are available from the corresponding author.
